# Dental Caries and Erosion in Children with Nephrotic Syndrome on Long-Term Liquid Medications—A Cross-Sectional Observational Study

**DOI:** 10.3390/jcm14248669

**Published:** 2025-12-07

**Authors:** Adel N. Radwan, Osama M. Felemban, Jameela A. Kari, Sherif M. El Desoky, Khlood Baghlaf, Heba Mohamed Elkhodhary

**Affiliations:** 1 King Abdulaziz University Dental Hospital, Jeddah 22252, Saudi Arabia; anradwan@kau.edu.sa; 2 Pediatric Dentistry Department, Faculty of Dentistry, King Abdulaziz University, Jeddah 21589, Saudi Arabia; omfelemban@kau.edu.sa; 3 Pediatric Nephrology Unit, Department of Pediatrics, Faculty of Medicine, King Abdulaziz University Hospital, King Abdulaziz University, Jeddah 21589, Saudi Arabia; jkari@kau.edu.sa (J.A.K.); sherifd68@hotmail.com (S.M.E.D.); 4 Department of Pedodontics and Oral Health, Faculty of Dental Medicine for Girls, Al Azhar University, Cairo 11765, Egypt; hebaelkhodary.2625@azhar.edu.eg

**Keywords:** caries, erosion, liquid medication, nephrotic syndrome

## Abstract

**Objectives**: This study aimed to evaluate the experience and consequences of dental caries and erosion in children aged 1–14 years on long-term liquid medications compared to those not on these medications. **Methods**: A cross-sectional observational study was conducted using a WHO-adapted questionnaire, medical surveys, and oral examinations. Participants included children with nephrotic syndrome in two groups: those on long-term liquid medications for at least three months (study group) and those not taking liquid medications (control group). The Decayed, Missing, and Filled Surfaces index (dmfs/DMFS) assessed caries, while the Pulpal Involvement, Ulceration, Fistula, and Abscess index (PUFA/pufa) measured caries consequences. The Basic Erosive Wear Examination index (BEWE) assessed erosion. **Results**: A total of 64 participants were included, with 33 in the study group and 31 in the control group. The study group had a significantly higher mean dmfs/DMFS of 16.9 ± 12.6 versus 5.2 ± 4.0 in the control group (*p* < 0.001). The PUFA/pufa index was also higher in the study group (0.8) compared to the control group (0.1) (*p* = 0.009). Erosion showed a non-significant increase with a BEWE score of 0.2 vs. 0.03 in the control group (*p* = 0.053). **Conclusions**: Long-term liquid medication use significantly affects dental caries in children after three months.

## 1. Introduction

Chronic illnesses during childhood have consistently been linked to a reduced quality of life for patients and their family members, as well as deterioration in family dynamics [1]. These children face not only the immediate stress of their illness but also ongoing and widespread stress due to the complexities of following detailed treatment plans and attending medical appointments. They frequently struggle with the difficulties of missing school and feeling different from their peers [2]. Furthermore, children with chronic illnesses often require liquid medications for extended periods [3]. It has been suggested that the prolonged use of these medications can increase their risk of developing dental caries [3]. This issue is often overlooked, as the primary medical condition tends to overshadow subtler aspects of the child’s overall health. Under these circumstances, parents typically concentrate on primary medical issues. Consequently, it is reasonable to expect a decline in oral hygiene as a child’s daily routine is disrupted [4].

Liquid medications can be categorized into several types, including solutions, suspensions, and syrups, among other forms. In these liquid formulations, the active pharmaceutical ingredient is either dissolved or dispersed within an appropriate vehicle. Although water is the most commonly used vehicle, it is mainly suitable for water-soluble drugs that possess a palatable flavor [5]. A significant obstacle in the administration of liquid medications to children is their inherently bitter flavor [6]. To enhance their acceptability, these medications are often modified by adding colors, flavors, and sweeteners to improve their palatability [6,7]. Using sweeteners is a common method for disguising flavors, as children can detect sweetness from a young age [5].

Sweeteners can be categorized into two primary classifications, natural and artificial [8]. Although synthetic alternatives such as sodium saccharin, sodium cyclamate, aspartame, and sorbitol are available, sucrose continues to be the preferred option in the pharmaceutical industry [6]. Sucrose is a disaccharide composed of glucose and fructose that naturally occurs in various fruits and vegetables. The commercial production of sucrose involves the processing of sugar cane or sugar beets [8]. The preference for sucrose is due to its affordability, antioxidant properties, capacity to preserve formulations, and ease of processing [6].

Pediatric liquid medications (PLMs) most often contain sucrose as a sweetening agent to mask the bitterness of active ingredients and make them more palatable; however, this may pose a risk factor to oral health. Sugar has been added to almost 50% of drugs in liquid form [6]. Therefore, awareness of the potential negative effects, including dental caries and/erosion from long-term usage of PLMs is important. Evidence regarding a relationship between sugary PLMs and dental caries and erosion in children with chronic conditions as nephrotic syndrome is limited. Therefore, it is imperative to conduct clinical studies to provide evidence on the potential negative effects of long-term consumption of sweetened PLMs among this group of patients and to increase the awareness among health care practitioners about this correlation. Therefore, this study aimed to assess the experience, severity, and consequences of dental caries, as well as dental erosion in a group of children aged 1–14 years, taking long-term liquid medications in comparison to those not on such medications.

## 2. Materials and Methods

### 2.1. The Study Design

The study followed a cross-sectional observational design and was reported following the STROBE checklist [9]. Ethical approval was obtained from the Research Ethics Committee at King Abdulaziz University Faculty of Dentistry (KAUFD) no. 042-02-23. The study aimed to evaluate dental caries and erosion in children on long-term medications. Children with nephrotic syndrome (NS) are often prescribed long-term liquid medications, as oral corticosteroids which contain sugars as additives [10], making them a suitable study group. Furthermore, some of the NS patients are prescribed medications in tablet form, depending on their age and ability to comply with treatment regimens. This group serves as an effective control for comparison purposes since they are suffering from the same systemic condition. Consequently, we opted to recruit the subjects from the pediatric nephrology at King Abdulaziz University Hospital, Jeddah, Saudi Arabia. Data collection was conducted between May 2023 and June 2024.

### 2.2. Participants

The research included children aged 1 to 14 years with a confirmed diagnosis of NS. Parents were required to speak either Arabic or English and could read. The research excluded children who have medical conditions requiring substantial modifications to their carbohydrate intake, including those with diabetes or obesity. Children with physical or cognitive impairments were also excluded. The participants were recruited during their regular follow-up appointments at the pediatric nephrotic departments. Following their nephrology team consultation, all patients who fit the inclusion and exclusion criteria were invited to participate in the study. Verbal explanations about the research idea, benefits, and risks were provided. Written informed consent was acquired from the guardians of all children who participated in the study.

### 2.3. Data Collection

Data collection encompassed medical history information and an oral clinical examination. The medical information was collected from the patients’ medical records and included data about their diseases and medications. The medical history of the subjects was recorded including the time of diagnosis of NS and any other relevant conditions. The information about prescribed medications was also recorded and included the name, type, duration, frequency, start date, and time of consumption of the medication that the child is currently taking or previously. Participants and their parents were also asked about post-medication oral hygiene practices, such as whether the participant drinks water, rinses, or brushes his/her teeth after taking the medication.

The final step in data collection involved a clinical oral examination by a single calibrated examiner wearing gloves, mask, and a gown, and conducting an assessment using a handheld flashlight and tongue depressors while the child was seated on a chair in the pediatric nephrology clinic. Examination was performed systematically, starting from the upper right last molar and proceeding sequentially through all the teeth to the upper left last molar, then from the lower left last molar to the lower right last molar. Dental caries was detected utilizing the dmfs/DMFS scoring system. This was recorded using the criteria outlined by the World Health Organization (WHO), for permanent and primary teeth [11]. The teeth were not air-dried, and plaque was not removed. The examiner looked for indications of tooth decay, such as white spot lesions, and recorded all types of restorations and crowns. Missing teeth were also recorded. All observations were noted based on the number of affected surfaces. In cases where proximal contact remained intact and no signs of decay were visible, no further investigation or radiographs were performed. The PUFA index evaluates the consequences of untreated dental caries by documenting conditions such as pulp exposure, ulceration, fistula, and abscess formation, without cleaning or excavating the decayed material [12]. The PUFA/pufa index was recorded to identify the progression and clinical outcomes of untreated carious lesions. Tooth erosion was evaluated using the Basic Erosive Wear Examination (BEWE) system, which quantifies erosion based on the extent of tooth structure loss. The BEWE scoring scale from 0 (no erosion) to 3 (hard tissue loss involving ≥ 50% of the surface) [13].

To establish inter-examiner consistency, the examiner assessed 10 patients (8 healthy and 2 with NS) prior to the data collection, recording dmfs/DMFS, PUFA/pufa, and BEWE scores. These patients underwent a follow-up examination after a two-week interval, resulting in an intraclass correlation of 0.994 (95% CI: 0.992–0.996) indicating high intra-examiner reliability. The data collected from these 10 participants were not incorporated into the main study sample.

Participants were divided into two groups. The study group consisted of nephrotic syndrome patients who had been using liquid medications for an extended period, with all participants meeting the criterion of ≥3-month duration criterion for liquid medication use [3,7]. In contrast, the control group was made up of patients who were either on tablet-form medications, had been using liquid medications for a shorter period of less than three months, or were not on any medications.

### 2.4. Sample Size

Based on the results of Goyal et al. 2019 [3], who found that the mean dmfs for NS patients was 7.5 ± 6.73 and the mean dmfs for the control group was 1.96 ± 3.03, it was assumed that the mean dmfs in the study group was 7 ± 6.5 and for the control group was 2 ± 3.5. Based on these assumptions, a minimum of 30 subjects in each group was required to detect a statistically significant difference between groups, with 95% power at the 0.05 significance level [3].

### 2.5. Statistical Analysis

Statistical data analysis was performed using Statistical Package for Social Science (IBM SPSS Statistics for Windows, version 20, IBM Corp., Armonk, NY, USA) software. The chi-square test and Fisher’s exact test were used to compare categorical variables between groups. *t*-test was used to compare continuous variables between groups. The level of significance was set at *p* < 0.05. Mann–Whitney U test was used to compare continuous data with non-normal distribution between groups. Two multiple linear regression models were modeled. The first regression model used the decayed component of the DMFS index as the dependent variable, while the second model used the PUFA index. In both models, the main independent variables were the long-term consumption of oral liquid medications (OLMs), and the covariates were age and father’s education level. These covariates were selected because they showed an association with both the exposure (OLM use) and the outcome variables in the bivariate analysis, making them potential confounders that warranted adjustment in the multivariate models. If two variables were found to be redundant or highly correlated, only one was included in the final model to prevent multicollinearity.

## 3. Results

### 3.1. Demographic Data

The total number of participants in this study was 64, with 33 in the study group and 31 in the control group. The age distribution differed significantly between the groups, with 22 children (66.7%) in the study group aged 1–9 years, compared to only 8 children (25.8%) in the control group (*p* = 0.001). Fathers in the control group had significantly higher educational levels than those in the study group (*p* = 0.009) (Table 1). The mean number of years since the diagnosis of NS was significantly lower (*p* = 0.003) in the study group (5.2 ± 3.0 years) compared to the control group (7.8 ± 3.4 years). Only 2 subjects in the study group had diagnoses other than NS compared to none of the subjects in the control group (*p* = 0.493).

Table 2 shows details about the medical status of participants. Upon data collection, it was found that most of the subjects were taking prednisone (Predo^®^, Al-Jazeera Pharmaceutical Industries (JPI), Riyadh, Saudi Arabia) as their main medication for treating NS. Table 2 presents detailed comparisons of medication-related practices between the groups. Most children were taking Predo^®^ 24 (72.7%) in the study group and 23 (76.7%) in the control group, with no significant difference between groups (*p* = 0.720).

#### 3.1.1. Dental Caries and Erosion

Table 3 shows the mean dmfs/DMFS index. The mean decayed component was significantly greater in the study group (13.9 ± 9.7) versus the control group (4.1 ± 3.6) (*p* < 0.001). The total dmfs/DMFS index mirrored these trends, being significantly elevated in the study group (16.9 ± 12.6) compared to the control group (5.2 ± 4.0) (*p* < 0.001). The PUFA/pufa index also revealed important differences between groups. The mean visible pulp component was significantly higher (*p* = 0.004) in the study group (0.5 ± 1.3) than in the control group (0.0 ± 0.0). The Basic Erosive Wear Examination (BEWE) scores were slightly higher in the study group (0.2 ± 0.6) compared to the control group (0.03 ± 0.2), but this difference was not statistically significant (*p* = 0.053).

**Figure 1:**
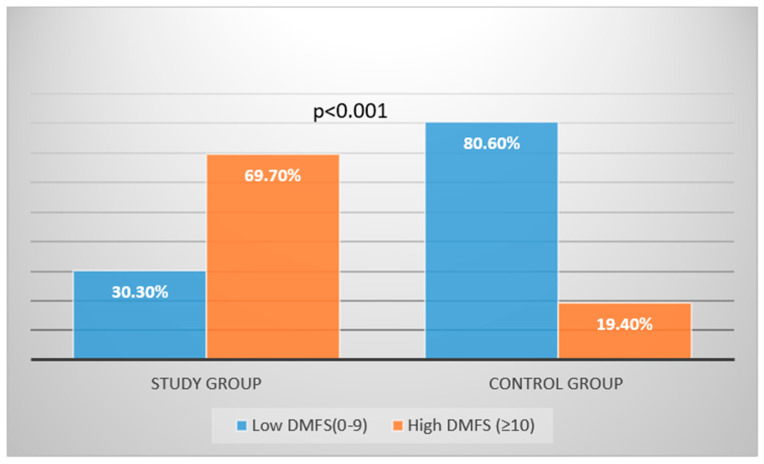
Caries distribution in the study and control groups.

The mean dmfs/DMFS scores were categorized as high (10 or more) or low (0–9) (Figure 1). In the study group, 69.7%had high scores, compared to 19.4% in the control group. Conversely, 30.3% of the study group had low scores, compared to 80.6% in the control group. Children in the study group were 3.6 times more likely to have high dmfs/DMFS (≥10) than those in the control group, and the increase in odds was statistically significant (*p* < 0.001).

#### 3.1.2. Logistic Regression

Table 4 presents the adjusted regression analysis results examining the associations between various independent variables and dmfs/DMFS scores. In the adjusted model for dmfs/DMFS, which explained 25.3% of the variance (R^2^ = 0.253), the study group continued to have significantly higher dmfs/DMFS scores compared to the control group (β = 7.0, 95% CI: 0.7–13.3, *p* = 0.029). However, the associations observed for age and father’s education in the unadjusted model were no longer statistically significant after adjustment. Older children (ages 10–14) had lower dmfs/DMFS scores, but the association was not significant (β = −3.8, 95% CI: −9.6–1.9, *p* = 0.187). Likewise, children whose fathers had a college education did not show a significant association with dmfs/DMFS scores after adjustment (β = −4.1, 95% CI: −10.9–2.7, *p* = 0.231). The variables related to the consumption of milk with sugar, sweet candy, or gum with sugar were not included in the adjusted model due to their lack of significance in the unadjusted analysis. The multiple linear regression model for the PUFA/pufa index yielded non-significant results regarding the association between long-term use of liquid medications and PUFA/pufa after controlling for age and father’s education.

## 4. Discussion

The study aimed to examine oral consequences of caries in children 1–14 years with NS who are on long-term liquid medication, compared to those not receiving such treatments. Information on diagnosis and medication was collected. In the current study, the dmfs/DMFS index, BEWE score, and PUFA/pufa index were used to assess dental caries, erosion, and consequences of untreated caries. The investigation identified a significant increase in the dmfs/DMFS score, along with the d/D component and PUFA/pufa index, in the study group compared to the control group. In contrast, the difference in BEWE scores between groups was not statistically significant.

An observational design was used, defining exposure as prolonged administration of liquid medication to patients. Dental caries and erosion were evaluated and compared between exposed and non-exposed individuals. While several departments were invited to participate, the pediatric nephrology department provided substantial resources, making children with NS patients ideal candidates for several reasons. Primarily, corticosteroids are the standard treatment for NS, and according to a study performed in 2015, Prednisolone has 1.9 g of sugar in every 5 mL of medication [14]. Additionally, the medication can be administered in liquid form, which can serve as the study group, while those taking tablets can function as a control group for comparison purposes.

The designation of a period of three months or more as long-term medication exposure was established to align with the studies by Goyal et al. in 2016 and 2019, where they defined long-term as three months or longer [3,7]. Furthermore, a 2022 study revealed that 16,401 chronic medications were prescribed to 3920 pediatric patients over a year, with these medications being prescribed for an average of 135 ± 61.0 days, equivalent to 4.5 months [15]. Additionally, the current study examined a medication for a chronic condition, and as previously mentioned, a condition is considered chronic if it persists for more than three months or is likely to last beyond three months [16].

The study group exhibited a significantly higher dmfs/Dmfs score (16.0 ± 12.6) compared to the control group (5.2 ± 4.0) (*p* < 0.001), suggesting that liquid medication contributes to increased dental caries. These findings are consistent with earlier research [3,7,17]. Sahgal et al. (2002) reported that a group of 51 children on liquid medication had notably higher dmft and dmfs scores compared to 54 children not on medication [17]. We gathered data on dietary habits and oral hygiene practices, analyzing them as potential confounding factors. However, we did not find significant confounding effects, and these results are not included in this manuscript.

These findings highlight the importance of providing families with support and guidance on the oral health risks associated with extended use of liquid medications. Effectively conveying dental health information can help prevent caries in both primary and permanent teeth. Targeted oral health promotion programs, including digital tools and teledentistry [18], can effectively support children and educate parents, especially in underprivileged populations with systemic conditions. Ensuring access to these resources can enhance preventive care and address disparities in oral health.

A case–control study by Stecksén-Blicks et al. (2004) identified a significant correlation between the duration of liquid medication and the dmfs value [19]. Research conducted by Goyal et al. in 2016 and 2019 produced findings consistent with our own [3,7]. In 2016, Goyal et al. studied children aged 2–12 years old diagnosed with epilepsy who were taking long-term liquid medication, and they found a 2.55 times higher risk of developing dental caries compared to those who were not using this type of medication [7]. In 2019, Goyal et al. 2019 examined 455 children with various chronic conditions on long-term liquid medication and found that the prevalence of caries was significantly higher than in a control group of 531 children who were not on those medications [3].

In the current study, evaluation of the PUFA/pufa index reflecting consequences of untreated dental caries showed a significantly higher occurrence of visible pulp involvement, fistula, and abscess in the study group compared to the control group. These findings highlight the complex interplay of factors influencing oral infection and inflammation, suggesting that while medication type may contribute to early-stage dental caries, its effect on advanced conditions may also depend on behavioral or clinical factors not captured in the current study. In contrast to the findings of the current study, Goyal et al. (2019) reported, after regression analysis, that children using non-fluoridated toothpaste had a 1.074 times higher risk of developing dental caries compared to those using fluoridated toothpaste [3]. In agreement with previous studies, our study identified various confounding factors, including age and paternal education [20], that differed significantly between the Study and Control Groups. Although younger age and lower paternal education initially correlated with higher dmfs/DMFS scores, these associations weakened and became statistically insignificant after adjusting for medication type. This suggests that their influence may be reduced when medication is taken into account.

Dental erosion was assessed using the BEWE index, and no significant differences were found between the study and control groups; thus, the null hypothesis was not rejected. This outcome may suggest that long-term use of oral liquid medications does not substantially contribute to dental erosion, or that the effects require a longer duration to become clinically evident. It is also possible that the sample size was insufficient to detect subtle differences or that the BEWE index may not be sensitive enough for detecting early erosive changes in this population. Additionally, while the difference in BEWE scores approached statistical significance (*p* = 0.053), it is important to acknowledge that the study may have been underpowered to detect differences in erosive wear.

Regression analysis of caries experience (dmfs/DMFS) showed that children on long-term liquid medications consistently exhibited higher scores than those taking tablets. Even after adjusting for confounding variables, this association remained statistically significant, suggesting a potential link between the prolonged use of syrup-based medications and increased caries burden. While younger age and lower paternal education initially appeared to be associated with higher dmfs/DMFS scores, these relationships weakened and lost statistical significance after adjustment, indicating that these factors may be less influential when accounting for medication type. In the regression model, the initial associations of younger age and lower paternal education with higher dmfs/DMFS scores weakened and became statistically insignificant after accounting for the sample size and group differences, suggesting that the form of medication may play a more decisive role in caries experience than these demographic factors. In contrast to the findings of the current study, Goyal et al. (2019) [3] reported different outcomes concerning confounding factors. They discovered after regression analysis that children using non-fluoridated toothpaste had a 1.074 times higher risk of developing dental caries compared to those using fluoridated toothpaste. Moreover, snacking between meals once a day increased the risk of dental caries by 3.08 times compared to not snacking at all. Furthermore, consuming sugary foods or drinks frequently throughout the week was associated with a 2.67 times greater risk of dental caries compared to not consuming them [3].

The primary strength of this study lies in the careful selection of the control group, which shared the same diagnosis, medication type, age range, and population as the study group, differing only in the type of medication they used. However, the control group was heterogeneous, consisting of children with less than three months of liquid medication use as well as those not currently taking any medication. This design allowed isolation of the effect of the prolonged liquid medication use. Another major strength of this research is its clinical relevance, as it addresses a largely neglected group that faces a high risk of dental decay and erosion.

This study has several limitations. First, the observational design prevents establishing a temporal relationship between exposure and outcomes, making it unclear whether dental caries and erosion developed before or after the medication use. Second, examiner blinding was not possible, which may have introduced observer bias, as the same examiner collected medication and diagnostic information from parents and verified it through patient records. Interproximal radiographs were not conducted, which could have resulted in false negatives in the diagnosis of dental caries. Additionally, the study’s relatively small sample size may affect the generalizability of the results, a limitation that was unavoidable since all eligible participants in the nephrology department were included. Finally, the broad age range of 1–14 years could have influenced caries outcomes, as children within this broad spectrum exhibit varying factors that affect dental caries.

Furthermore, future research and clinical practice could consider developing liquid medications that avoid sucrose, as noted in the Introduction. Sucrose acts as a substrate for cariogenic bacteria such as Streptococcus mutans and, compared to tablet forms, liquid medications remain in contact with teeth for longer periods, increasing bacterial activity and enamel demineralization. Exploring alternative sweetening agents that maintain palatability while minimizing cariogenic potential could represent an essential step in reducing the oral health risks associated with long-term pediatric liquid medication use.

## 5. Conclusions

The study identified a clear association between dental caries and the prolonged use of liquid medications in children with NS. The dmfs/DMFS score, the d/D component and PUFA/pufa index were significantly higher in the study group compared to controls. Despite the presence of numerous potential confounding factors, none significantly influenced the study’s results. It is important for pediatricians to be aware of this information before prescribing long-term liquid medications, especially those containing sugar, to consider alternatives or at least advise on oral hygiene practices and regular dental check-ups. Additionally, pediatric dentists can utilize this information to identify and manage caries risk in children using long-term liquid medications. Although these findings can be extended to other chronic pediatric conditions involving liquid medications, further research is needed for each disease category, as some diseases or medications may directly impact oral health, or be affected by it in return.

## Figures and Tables

**Table 1:** The demographic characteristics of the children included in the study.

Demographics		Study Group *n* = 33	Control Group *n* = 31	*p*-Value
**Gender**	Male	19 (57.6)	16 (51.6)	0.632 €
	Female	14 (42.4)	15 (48.4)	
**Age**	Mean ± SD	7.6 ± 3.1	10.7 ± 3.5	<0.001 * †
**Father** **education**	Less than high school	10 (30.3)	3 (9.7)	0.009 * €
	High school	11 (33.3)	10 (32.3)	
	College	8 (24.2)	18 (58.1)	
	I don’t know	4 (12.1)	0	
**Mother education**	Less than high school	5 (15.2)	2 (6.5)	0.440 €
	High school	14 (42.4)	10 (32.3)	
	College	13 (39.4)	18 (58.1)	
	I don’t know	1 (3.0)	1 (3.2)	

€ Chi-square test, † Independent sample *t*-test, * statistically significant (*p* < 0.05).

**Table 2:** Comparisons of medication-related behaviors.

Predo^®^		Study Group *n* = 33 *n* (%)	Control Group *n* = 30 *n* (%)	*p*-Value
**Currently or previously taking Predo^®^**	Current	24 (72.7)	23 (76.7)	0.720 €
	Previous	9 (27.3)	7 (23.3)	
**Frequency of** **Daily use**	2 or more/day	0	0	0.102 €
	Once/day	20 (60.6)	12 (40.0)	
	EOD	13 (39.4)	18 (60.0)	
**Onset of Predo^®^**	Less than 36 months	13 (39.4)	3 (10.0)	0.009 * F
	36 months or more	20 (60.6)	27 (90.0)	
**Duration of Predo^®^**	Less than 36 months	16 (48.5)	8 (26.7)	0.075 €
	36 months or more	17 (51.5)	22 (73.3)	
**Daily timing of taking Predo^®^**	Morning	3 (9.1)	5 (16.7)	0.146 F
	Afternoon	24 (72.7)	24 (80.0)	
	Evening to Bedtime	6 (18.2)	1 (3.3)	
**Brush teeth after taking Predo^®^**	Yes	0	0	0.493 F
	Sometimes	2 (6.1)	0	
	No	31 (93.9)	30 (100)	
**Drink water after taking Predo^®^**	Yes	16 (48.5)	25 (83.3)	0.016 * F
	Sometimes	7 (21.2)	2 (6.7)	
	No	10 (30.3)	3 (10.0)	
**Rinse mouth after taking Predo^®^**	Yes	2 (6.1)	2 (6.7)	0.883 F
	Sometimes	3 (9.1)	4 (13.3)	
	No	28 (84.8)	24 (80.0)	
**Taking Medications other than Predo^®^**	Yes	8 (24.2)	4 (12.9)	0.341 F
	No	25 (75.8)	27 (87.1)	
**Type of Medications other than Predo^®^** **(*n* = 12)**	Syrup	6 (75.0)	0	0.061 F
	Tablet	2 (25.0)	4 (100)	

EOD: Every other day, € chi-square test, F Fisher Exact test, * statistically significant (*p* < 0.05).

**Table 3:** Mean dmfs/DMFS, PUFA/pufa, and BEWE index scores.

Parameter	Study Group (*n* = 33)	Control Group (*n* = 31)	*p*-Value
**dmfs/DMFS Index**			
Decayed	13.9 ± 9.7 (Median = 13.0; Range = 0–56)	4.1 ± 3.6 (Median = 4.0; Range = 0–14)	<0.001 * §
Missing	2.6 ± 6.1 (Median = 0; Range = 0–25)	0.32 ± 1.2 (Median = 0; Range = 0–5)	0.077 §
Filled	0.4 ± 1.1 (Median = 0; Range = 0–4)	0.8 ± 1.5 (Median = 0; Range = 0–7)	0.044 * §
Total dmfs/DMFS	16.9 ± 12.6 (Median = 14.0; Range = 0–56)	5.2 ± 4.0 (Median = 5.0; Range = 0–14)	<0.001 * §
**PUFA/pufa Index**			
Visible pulp involvement	0.5 ± 1.3 (Median = 0; Range = 0–5)	0 ± 0 (Median = 0; Range = 0–0)	0.004 * §
Ulceration	0 ± 0 (Median = 0; Range = 0–0)	0 ± 0.4 (Median = 0; Range = 0–2)	0.302 §
Fistula	0 ± 0.4 (Median = 0; Range = 0–1)	0 ± 0 (Median = 0; Range = 0–0)	0.025 * §
Abscess	0 ± 0.3 (Median = 0; Range = 0–1)	0 ± 0 (Median = 0; Range = 0–0)	0.047 * §
Total PUFA/pufa	0.8 ± 1.8 (Median = 0; Range = 0–7)	0 ± 0.4 (Median = 0; Range = 0–2)	0.009 * §
**BEWE Score**			
BEWE Score	0.2 ± 0.6 (Median = 0; Range = 0–2)	0.03 ± 0.2 (Median = 0; Range = 0–1)	0.053 §

§ Mann–Whitney U test, * statistically significant (*p* < 0.05).

**Table 4:** The unadjusted and adjusted regression analysis of the dental caries scores dmfs/DMFS and confounding variables.

Independent Variables		Dependent Variable dmfs/DMFS Adjusted R^2^ = 0.253		Dependent Variable PUFA/pufa Adjusted R^2^ = 0.173	
		β 95% CI	*p*-Value	β 95% CI	*p*-Value
**Group**	**Study**	7.0 (0.7–13.3)	0.029 *	0.01 (−0.8–0.8)	0.979
	**Control**	Reference		Reference	
**Age**	**Older (10–14)**	−3.8 (−9.6–1.9)	0.187	−0.6 (−1.3–0.1)	0.107
	**Younger (1–9)**	Reference		Reference	
**Father education**	**College**	−4.1 (−10.9–2.7)	0.231	−0.5 (−1.3–0.4)	0.291
	**High school**	−1.5 (−8.3–5.3)	0.661	0.03 (−0.8–0.9)	0.067
	**<high school**	Reference		Reference	

* Statistically significant (*p* < 0.05).

## Data Availability

Data is available upon reasonable request for the authors.

## Abbreviations

The following abbreviations are used in this manuscript:

NS	Nephrotic Syndrome
PLM	Pediatric liquid medications
Predo^®^	Prednisone
EOD	Every other day
dmfs	Decayed, missing, filled surfaces
BEWE	Basic Erosive Wear Examination
